# Perspectives on waiting times in an antenatal clinic: A case study in the Western Cape

**DOI:** 10.4102/hsag.v26i0.1513

**Published:** 2021-04-30

**Authors:** Justine C. Baron, Doreen Kaura

**Affiliations:** 1 Department of Nursing and Midwifery, Faculty of Medicine and Health Sciences, Stellenbosch University, Cape Town, South Africa

**Keywords:** waiting time, barriers to waiting times, factors influencing waiting times, waiting time in antenatal clinic, facilitators of waiting times

## Abstract

**Background:**

Antenatal care (ANC) is vital in reducing maternal and neonatal morbidity and mortality. Globally, 85% of women had one ANC visit with a skilled birth attendant and only 58% received the recommended four ANC visits. Long waiting times (LWTs) in the antenatal clinic affects the utilisation of the service. Long waiting times are viewed as a significant barrier to ANC utilisation and needs further investigation.

**Aim:**

The aim of this study was to explore and describe the contextual realities within the antenatal clinic that influenced waiting times (WTs).

**Setting:**

This study was conducted in an antenatal clinic, within a Midwife Obstetric Unit (MOU), Western Cape, South Africa.

**Methods:**

This study utilised a qualitative methodology with a single case study design with three embedded units of analysis. Purposive sampling was used to recruit the participants. Data were collected through unstructured observation and semi-structured interviews with pregnant women and midwives. Interviews were audio recorded, transcribed and analysed using the framework method.

**Results:**

The antenatal clinics had LWTs. The barriers to WTs were related to staff factors, patient factors, operational factors, communication, equipment and infrastructure and other research participant recruitment.

**Conclusion:**

The factors that influenced WTs are multifaceted and interrelated. Many of the factors influencing the WTs could be remedied by implementing appropriate workflow strategies, improving communication and increasing equipment availability. The findings can be used to develop waiting time guidelines and improve WTs in the antenatal clinic.

## Introduction

Approximately, 303 000 women died in 2015 because of pregnancy and childbirth-related causes globally, with South Africa reporting a maternal mortality rate of 119 per 100 000 lives in 2017 (Knoema 2020; World Health Organization 2016:1). Antenatal care (ANC) is essential in preventing maternal and neonatal morbidity and mortality through the detection and treatment of pregnancy complications, the identification of high-risk women and referrals to appropriate levels of care (World Health Organization 2016:1). Long waiting times (LWTs) is one of the barriers to ANC uptake (Kyei-Nimakoh, Carolan-Olah & Mccann 2017:7). Long waiting times decreases a woman’s perception of satisfaction, negatively influencing her ANC attendance (Adeyinka et al. 2017:5). Thus, identifying the barriers to LWT could improve access to ANC services.

Antenatal care is the provision of care to pregnant women, by skilled healthcare professionals to ensure optimal health during pregnancy (World Health Organization 2016:1). Failure to receive ANC resulted in 50 million women suffering from maternal morbidities because of complications of pregnancy, which could have been avoided (Gupta et al. 2015:89). Globally, 85% of women had one ANC visit and only 58% received four ANC visits (Basha 2019:1). In South Africa, ANC coverage of one visit before 20 weeks gestation was 65.2% (Ntloana, Mazanderani & Sherman 2017:72).

Various challenges are attributed to poor ANC attendance, which includes lack of transportation, midwives’ attitude, sociocultural issues and LWTs (Abimbola et al. 2016:962). In South Africa, a waiting time (WT) of more than 2 h, in a primary care facility, is viewed as LWT (National Department of Health 2015:8). Many studies have reported LWTs within the antenatal clinic. In Kazakhstan, Central Asia, the average WT was 35.7 min and ranged between 0 and 300 min (Dauletyarova et al. 2018:4). In Nigeria, the average time in the antenatal clinic was 3.8 h and ranged from 1 to 7 h (Nwaeze et al. 2013:25).

The barriers to WTs are specific to the environment in which they occur and the type of work that is conducted in the facility. However, there are common threads that are related to scheduling, patient flow, resource allocation, inefficient infrastructure, poor communication, shortage of staff, operational planning, inexperienced staff, lack of equipment, slow speed of staff and patient characteristics (Baker 2017:58–59; Hong et al. 2013:31–37).

No studies were found on the factors that influence WTs in the antenatal clinic. Furthermore, reliable data on patient WTs in the antenatal clinic are not specified in the Western Cape of South Africa. Knowledge of the factors that contribute to WTs in the antenatal clinic can be used to reduce the WTs. Accurate measurement of WTs can also be used to inform WT guidelines. The reduction in WTs in the antenatal clinic could contribute to women being more likely to attend the ANC services.

The study intended to explore and describe the contextual realities within the antenatal clinic that influenced WTs. The aim was achieved through describing the WTs and exploring the perceived barriers to WTs.

## Research method

A qualitative methodology with a single type 2 case study approach was used (Yin 2014:70). The case was the antenatal clinic contained within a midwife obstetric unit (MOU). The approach included three units of analysis which were WTs within the antenatal clinic, the pregnant women attending the clinic and the midwives working in the clinic.

### Ethical considerations

The research was approved by the Health Research Ethics Committee of a University in the Western Cape and the Western Cape Provincial Department of Health, research division.

### Trustworthiness

Trustworthiness was ensured through data triangulation. Further, data saturation was reached during data collection and a further two interviews were conducted to confirm data saturation. Member checking was done during data collection as well as after data analysis. The transcripts were compared to the audio recordings to ensure consistency. Purposive sampling was used and during the interviews open ended questions with probes were used to ensure that thick, rich data was obtained.

### Setting

The study was conducted in the antenatal clinic within an MOU in the Western Cape. The antenatal clinic was chosen because of the high number of clients and the indications of LWT. The MOU is a primary care facility situated on the premises of a Community Health Centre (CHC). The MOU provides antenatal, intranatal and postnatal services to the low-risk women.

### Study sample

The MOU serves a middle- to low-income community that is predominantly (88%) Xhosa speaking (Statistics South Africa 2020). Each month, approximately 250–280 pregnant women attend the clinic for their initial ANC visit and approximately 1200–1300 women attend the clinic for their follow-up appointments. The study sample consisted of 14 participants, of which 12 were pregnant women aged between 18 and 46 years old (Table 1). Four midwives were working in the clinic and two declined to participate in the study.

**TABLE 1:** Demographic details of the participants.

Participants	Age	Gravidity and parity	Gestational age
Participant 001	38	G4P3-1 (neonatal death)	19 weeks
Participant 003	21	G1 P0	40 weeks
Participant 004	24	G1 P0	38 weeks
Participant 005	23	G2 P1	13 weeks
Participant 006	31	G3 P2	24 weeks
Participant 007	18	G1 P0	37 weeks
Participant 008	31	G3 P2	35 weeks
Participant 009	21	G1 P0	Undetectable uterus
Participant 011	46	G6 P5	20 weeks
Participant 012	33	G3 P1 M1	Undetectable uterus
Participant 013	25	G2 P1	4 weeks
Participant 014	19	G1 P0	Undetectable uterus
**Midwives**		-	-
*Experience*		-	-
Participant 002	20 years	-	-
Participant 010	4 years	-	-

### Data collection

Data were collected through unstructured observation and semi-structured interviews. Two semi-structured interview guides were used: one for the midwives and one for the pregnant women. The interview questions were related to the participants’ experience of WTs and their perceptions of the causes of WTs. Similarly, the midwives’ questions were related to their perceptions of the WTs and the causes of the WTs.

Data triangulation ensured that the perspectives on the barriers to WTs increased the validity of the findings (Yin 2014:152). All the participants could speak English fluently and chose to do the interview in English.

Pregnant women who had their initial visits and follow-up visits were recruited between 06:45 and 07:00. Written informed consent was obtained from the participants. Thereafter, the researcher followed the participants through their ANC visit. An unstructured observation of all the women was conducted. During the observation, the researcher wrote field notes of the WTs, the activities and procedures that the women underwent and the duration of the consultation and contact times. One participant was observed and interviewed per day because of the intensity of the observation. Interviews were conducted after the ANC visit in a private room within the MOU. The midwives’ interviews were conducted at their discretion. The interviews lasted between 30 and 60 min. Data saturation was reached after 10 interviews with the pregnant women.

### Data analysis

Data analysis was conducted using the framework method (Gale et al. 2013:4–5). The procedure for data analysis was as follows:

- Transcription: The audio recordings were transcribed by a professional transcriptionist. The transcriptionist completed a confidentiality report to maintain the integrity of the research.
- Familiarisation: The audio recordings were compared with the transcripts to ensure the accuracy of the transcripts and to become familiar with the data.
- Coding: Transcripts were read line by line and a code or codes were applied to the data.
- Developing a working analytical framework: After coding of the first few transcripts, a set of codes were decided on and applied to the rest of the transcripts. Where new information was found, it was coded, and the code was added to the list of codes.
- Applying the analytical framework: Each code was assigned a number so that the full names of the codes did not need to be written out. The subsequent transcripts were categorised using the existing codes.
- Charting data into the framework matrix: The data were inserted into a spread sheet, one for the pregnant women and one for the midwives and analysed separately. The data were categorised according to WTs and barriers to WTs.
- Interpreting the data: The findings were interpreted and placed into context according to the study’s objectives.

### Observational data

Each participant’s patient flow and their WTs were described. A table was created to map each participant’s patient flow and the activities they underwent. The WTs were categorised into contact time, WT and total time spent at the facility. The WT was calculated by deducting the contact time from the total time spent at the facility.

The field notes were analysed and the similarities, with the participants’ perspectives, were highlighted and used to validate the participants’ perspectives.

## Results

A description of the WTs in the antenatal clinic is provided here. The main themes that emerged from the data, related to the barriers to WTs, include staff factors, patient factors, operational functioning, communication, equipment and infrastructure and other research study participant recruitment.

### Waiting times in the antenatal clinic

Participants with initial visits experienced longer WTs, ranging from 338 to 503 min (Table 2), whilst participants with follow-up visits experienced WTs ranging from 105 to 216 min (Table 3).

**TABLE 2:** Initial visit waiting times.

Participant 008	Participant 009	Participant 011	Participant 012	Participant 013	Participant 014
**Contact time**					
54 min	66 min	135 min	65 min	88 min	191 min
**WT**					
284 min	371 min	368 min	354 min	363 min	301 min
**Total time at facility**					
338 min	437 min	503 min	419 min	451 min	492 min

WT, waiting times.

**TABLE 3:** Follow-up appointment waiting times.

Participant 001	Participant 003	Participant 004	Participant 005	Participant 006	Participant 007
**Contact time**					
30 min	9 min	12 min	16 min	14 min	17 min
**WT**					
151 min	154 min	105 min	149 min	216 min	172 min
**Total time at facility**					
181 min	163 min	117 min	165 min	230 min	189 min

WT, waiting times.

### Barriers to waiting times

#### Staff factors

The antenatal clinic was staffed with midwives, human immunodeficiency virus (HIV) counsellors and nurses. Staff shortage negatively influenced WTs:

‘So, you just can see if we have only three sisters and we have a lot of patients that day, […] They have to wait long for there’s nothing really we can do. Otherwise if we have four sisters at least, […] it’s quick, quick. But if we are three, we try but it’s not like when we are four.’ (Participant 010, midwife, female)

‘I think it’s … the shortage of staff. I don’t know [*Laughs*].’ (Participant 011, initial visit)

On Thursdays, a doctor from a referral hospital has appointment with high- risk patients. If the doctor is delayed at the hospital and arrives at the clinic late, the patients’ WTs will be extended:

‘So, she doesn’t really have waiting time unless maybe she had come, to via Mowbray and then they will delay her there and then she comes here late. Then they must wait for her because there’s nothing we can do.’ (Participant 010, midwife, female)

Staff arrival times and operational times were stated as reasons for WTs. The pregnant women indicated that the staff arrived on duty late and did not start working as soon as they arrived:

‘So, I was here at half past six. Guess what? They haven’t arrived at half past six. […]. So, I had to be in the queue. So, then they came in at eight, maybe … ja [*yes*].’ (Participant 008, 31, initial visit)

The women felt that the staff were working at a slow pace and staff conversations during consultations contributed to WT:

‘I think they are slow or maybe the patient needs attention maybe.’ (Participant 007, 18, follow-up visit)

‘Because they [*staff*] gossip a lot. Seriously, they gossip a lot.’ (Participant 007, 18, follow-up visit)

‘During a midwife’s consultation, another staff member interrupted the consultation, and a conversation was held during the patient’s consultation time. This lengthened the patient’s consultation time and increased the after coming patients waiting times.’ (Researcher’s observation, female, midwife)

Staff attitude and behaviours were seen to contribute to extended clinic visits. Participants felt that complaints are disregarded and therefore it was not worthwhile to complain. Others felt that complaints result in punishment, such as being seen last:

‘Sometimes we as patients we keep on back chatting with them […] they will tell you that okay you’re back chatting me, you better stay there, and you will be the last one.’ (Participant 009, 21, initial visit)

One participant mentioned that staff meetings during clinic operation times influenced WTs. The meetings were arranged for the late morning/afternoon, so that the follow-up patients could be seen. If there were women who arrived late, they would be seen after the staff meeting:

‘I think they were busy talking. […] But it is something serious for the staff. Yes. Maybe they were telling them what to do and whatsoever […]. So, we had to move from there [*midwife consultation room*] to there [*counselling room*] because maybe we were going to disturb whatsoever. So … Some sort of a small meeting.’ (Participant 014, 19, initial visit)

‘The above-mentioned meeting was related to an audit that was conducted by an external party. There were also less midwives attending to the women, on this day, as one midwife was on the walk-about with the auditor.’ (Researcher’s observation, female, midwife)

‘[…] But we normally try to shove meetings on Fridays. It depends. We normally … If it’s our staff meeting, only staff meeting, we normally do it, try to do it after clinic. We try and push the patients and then … But, you know, we sometimes get here late so those ones must wait. Otherwise the ones that we’ve got here we normally push them and then we do our meeting after. Because our staff meetings normally start around 11, 12.’ (Participant 010, midwife, female)

#### Patient factors

The number of patients influenced WTs. Patient numbers, per day, were inconsistent. Some days the clinic had few patients and other days there were many patients. The amount of follow-up visits and initial visits per day influenced the WTs:

‘Because most of the time they take a lot of the follow-ups. Today was a few for bookings and a lot for follow-ups.’ (Participant 014, 19, initial visit)

‘Today we were not many like some other days. Today it was short but before it was long.’ (Participant 003, 21, Follow-up visit)

The women who came for follow-up appointments arrived late or their arrival times were sporadic. They were not deferred to different days. This affected the WTs for the patients who came for their initial visits:

‘The follow-ups just come bit by bit we can see that it’s quiet. When you come in the morning, no man it’s not full. But one by one you will see them they come late.’ (Participant 002, midwife, female)

#### Operational functioning

The biggest contributor to WT was that follow-up visits were seen before the women who arrived for their initial visits. The initial visit appointments also had more activities that need to be completed, which contributed to the time spent at the facility:

‘It’s not that I waited long because they started with those ones [*follow-ups*], that’s the problem. They are not mixing. If you are booking, they are starting with those ones, the old ones.’(Participant 006, 31, Follow-up visit)

‘[…], [*W*]e do we start with the follow-ups. Because the first-time bookers there’s a lot to be done.’ (Participant 002, midwife, female)

There was no consistent patient flow in the clinic which contributed to the patients’ WTs (Tables 4 and 5). The order in which the women arrived was lost as the women shifted seats to enter into the different rooms. Many of the women for their initial visits felt disorientated with the clinic’s flow:

**TABLE 4:** Follow-up visits patient flow.

Time	Participant 001	Participant 003	Participant 004	Participant 005	Participant 006	Participant 007
06:00	06:45 Arrival time	-	-	06:00 Arrival time	-	-
07:00	-	07:05 Arrival time 07:08–07:12 Urine analysis Weight	07:00 Arrival time 07:10–07:14 Urine analysis Weight 07:33 Blood pressure (machine needs charging) 07:53–07:54 Blood pressure	07:05–07:07 Urine analysis Weight 07:52–07:53 Blood pressure (machine removed)	07:13 Arrival time	7:05 Arrival time 07:13–07:16 Urine analysis Weight
08:00	08:58–09:10 U/S (Ultrasound) consult	08:50–08:51 Blood pressure	08:49–08:55 Midwife consult 08:57 Reception	08:19–08:21 Blood pressure 08:30–08:40 Midwife consult (results) 08:45 Reception	-	08:46–08:48 Blood pressure
09:00	09:28–09:46 Midwife consult (telephone not available) 09:47 Reception	09:42–09:48 Midwife consult 09:48 Reception	-	-	-	-
10:00	-	-	-	-	10:34–10:45 Midwife consult (results) 10:46–10:49 Reception	10:01–10:10 Midwife consult 10:11–10:14 Reception

**TABLE 5:** Initial visits patient flow.

Time	Participant 008	Participant 009	Participant 011	Participant 012	Participant 013	Participant 014
05:30	-	-	05:50 Arrival time	-	-	-
06:00	06:30 Arrival time	06:20 Arrival time	-	06:10 Arrival time	06:00 Arrival time	06:28 Arrival time
07:00	07:34–07:41 Urine analysis Weight Height	-	-	-	-	
08:00	-	08:39–08:46 HCT (HIV counselling and testing) counselling	08:17–08:35 History taking MUAC (mid upper arm circumference) 08:47–08:53 Urine analysis Weight Height	08:44–08:47 HCT counselling 08:53–08:57 Urine analysis Weight Height	08:26–08:32 Urine analysis Weight Height 08:49–09:09 Reception	08:37–08:41 HCT counselling 08:48–08:52 Reception 08:56–09:06 Urine analysis Weight Height
09:00	09:06–09:19 Reception 09:22–09:25 Blood pressure HB 09:30–09:36 HIV counsellor room 90:36–09:36 BP room	09:11–09:15 Urine analysis Weight Height 09:25–09:29 History taking MUAC	09:04–09:10 HCT counselling	09:10–09:31 Reception 09:37–09:39 Blood pressure Hb (haemoglobin)	09:39–09:42 Blood pressure Hb	-
10:00	10:10–10:11 Counselling room 10:40–10:44 History taking MUAC 10:44–10:52 Counsellors room	10:15–10:30 Reception 10:47–10:48 Seminar room Hand in folder	10:11–10:42 Reception	10:15–10:30 History taking	10:49–11:02 Seminar room History taking MUAC	-
11:00	11:56–12:08 Midwife consult	11:00–11:03 Blood pressure Hb	11:34–11:37 Bloods drawn 11:45–11:50 Blood pressure Hb	11:15–11:20 Blood taken	11:28–11:44 Bloods drawn	11:20–11:28 History taking 11:28–11:31 HIV counsellor room11:40–12:01 Study recruiter
12:00	-	12:03–12:05 Blood taken 12:34–12:39 Hand in blood at seminar room	-	12:04–12:08 Hand in blood at seminar room 12:57–13:09 Midwife consult	12:24–12:28 Hand in blood at seminar room	12:35–12:43 Bloods drawn 12:50–12:53 Hand in blood at seminar room 12:54–14:17 Study recruiter
13:00	-	13:12–13:37 Midwife consult	13:00–13:11 Hand in blood at seminar room 13:17–13:59 Recruited for a study	-	13:17–13:31 Midwife consult	-
14:00		-	14:00–14:13 Midwife consult	-	-	-

HIV, human immunodeficiency virus; HCT, HIV counselling and testing; MUAC, mid upper arm circumference; Hb, haemoglobin; BP, blood pressure.

‘[…] Some of them they started with history room and they came back to diabetics and then after that they went to counsellors so we just as I say it’s not one routine that we have to do like this.’ (Participant 009, 21, Initial visit)

‘Maybe a few will go to counsellors, others will go to history taking but there are a lot. […] There’s no pattern at all.’ (Participant 010, midwife, female)

‘Patients were seen asking one another which rooms to go to next. Two patients were seen running to get to a room first so that their blood pressure could be taken.’ (Researcher’s observation, female, midwife)

Antenatal care activities also influenced WTs. Failure to capture all the patient’s details resulted in the patient being sent back to complete their maternity case records (MCRs). Similarly, if the work activities were not done, the midwife did not have all the required information for her consultation:

‘[…] Remember I went twice there because the first time they forgot to put my diabetic’s details on the portfolio.’ (Participant 009, 21, Initial visit)

‘[…] Because they can also strengthen the waiting period because we will call the new booker and then you find out that maybe blood pressure is not yet done.’ (Participant 010, midwife, female)

During the midwives’ consultations managing complications, such as pregnancy-induced hypertension, sexually transmitted diseases and HIV influenced WTs for the after coming patients. The timing of the occurrence of the complications had an effect on the WTs:

‘And if the blood pressure it’s high, it’s high, we need to do something. […] So, once that you see that the patient is a severe, having severe high blood pressure then you must stop everything and attend that patient. So, the ones … the others have to wait. […] And if maybe you’re number one or you’re number two is having a problem then everyone must wait. But it’s better if it’s number last that is having a problem [*laughs*] because you’re almost done with everyone […].’ (Participant 010, midwife, female)

Telephonic referrals caused delays with the midwife consultation. The lengthened consultation time influenced WTs of the after coming patients. The midwife also experienced delays with the referral hospital where the accepting doctor needed confirmation from a senior physician before accepting the referral:

‘And maybe we must call to Groote Schuur [*referral hospital*] and then sometimes you know you can wait on the line for 15 minutes waiting for Groote Schuur sisters to maybe come and give you whatever date that the patient must go there on. […] So, she … Because the doctor took orders and then he was like, okay, just hold on sister, I must go and discuss the patient with my senior.’ (Participant 010, midwife, female)

During patient consultation, completing the patients’ documentation is time-consuming. The duplicate documentation lengthens the consultation times, which influences the WTs:

‘And also, paperwork is too much, yo! […] What you wrote in MCR we must write again in the thingy, in the form that you must place in the folder. […] And then the MCR book is missing, so there is nothing to back you up that you did whatever you did.’ (Participant 010, midwife, female)

A midwife felt that students, especially the third-year midwifery students, contributed to WT. The procedures need to be explained to the students and the findings need to be confirmed by the midwife:

‘They sometimes delay us because in the middle you must teach, you must explain. So, it also takes time.’ (Participant 010, midwife, Female)

‘The student done an abdominal palpation and symphysis-fundus measurement. Thereafter, the midwife repeated the procedure to confirm the student’s findings. The consultation times where students were present were longer.’ (Researcher’s observation, female, midwife)

#### Communication

Communication was a challenge as a few of the participants were foreign nationals and did not understand Xhosa. The staff mainly spoke Xhosa unless they were approached, and the patient informed them that they did not understand the language. The lack of understanding and poor communication resulted in the women feeling lost and confused. One of the participants acted as a translator for the staff and this delayed her ANC activities. The language barrier resulted in the women waiting, and then seeking assistance, which affected their WTs:

‘As you know, if you are a foreigner or if you can’t speak Xhosa and you can’t understand them you can take so long. I think that is another problem because they didn’t speak English. […]. They only speak Xhosa and it was really necessary then, so I didn’t understand till I asked someone that was next to me […].’ (Participant 004, 24, Follow-up visit)

‘They just call me for other lady behind because she don’t speak any English and she don’t understand English, so they’re calling me to make her straight.’ (Participant 005, Follow-up visit)

‘A foreign national woman seemed lost and stood in the clinic looking around. Thereafter, she asked another patient what she needs to do and where she needs to go to.’ (Researcher’s observation, female, midwife)

#### Equipment and infrastructure

Equipment integrity and availability negatively impacted on WTs. In the clinic, one nurse would take the patients’ blood pressure. The one baumanometer influenced how long it took to assess the patients. Similarly, another participant had to wait as a result of a telephone that was not available because the birthing unit and the antenatal clinic shared a telephone:

‘It was low because it’s just going off when I was busy doing it. They you wait it will be off. Because I didn’t sit like 15 minutes waiting, maybe five to ten minutes then they called me again to do the blood pressure.’ (Participant 004, 24, Follow-up visit)

‘So, ja [*yes*]. And they must do blood pressures and there is only one machine. There is only one nurse. So, we have … we also wait for them.’ (Participant 010, midwife, Female)

‘The baumanometer was seen being removed from the antenatal clinic and at a later stage the women’s blood pressures were repeated.’ (Researcher’s observation, female, midwife)

‘I came back, they send me back, to the clinic, to take another date for appointment-Groote, Groote Schuur. Then I wait there by the nurse there by the clinic. I think 10 minutes because there was no phone to call there by Groote Schuur …’ (Participant 010, midwife, female)

‘The above participant waited 18 minutes as the telephone was not available to make an appointment.’ (Researcher’s observation, female, midwife)

There were no resting beds for hypertensive patients in the antenatal clinic. The patients needed to be taken and managed further in the labour ward by the midwives in antenatal clinic. The labour ward and the antenatal clinic are on opposite sides of the MOU. Walking between the two units was time-consuming and also waiting to use the telephone, if it was in use. This influenced the WT of the after coming patients:

‘It stops you and you don’t have resting beds in antenatal clinic. You need to stop, you need to leave, you need to go to labour ward and ask a bed there for your patient to rest while waiting for the ambulance. […] So, you must go to labour ward, your patients must wait. And then by the time you get there the sister is already on your line discussing the patient, so you must sit and wait for the phone. So, we’re using one phone […] It’s far from the labour ward to clinic.’ (Participant 010, midwife, female)

The antenatal clinic has four patient toilets of which only two were usable. There was one nurse doing the urine analysis and measuring women’s weight and height. This resulted in queues at the toilet:

‘Yes, also because the toilet’s only two that work, and the others are full of equipment, the other toilets for the patients. […] So, if they call four and they call three of us then we have to wait for the queue there, and there’s only one person also testing the urine in the waiting room.’ (Participant 014, 19, initial visit)

On Thursdays, when the doctor was at the antenatal clinic, the doctor used a midwife’s cubicle. There would thus only be three midwives attending to the follow-up appointments and initial visits. The fourth midwife would assist the doctor. This influenced the WTs as fewer midwives were attending to the patients:

‘What we normally do is on Thursdays if we know that we’re having doctor’s clinic we don’t book a lot of patients for that day because we know one sister is going to … her cubicle is going to be used by doctor and then we’re going to be three sisters.’ (Participant 010, midwife, female)

#### Other research participant recruitment

Another study was conducted at the MOU during data collection. Two of the participants were recruited for the other study. One of the participants felt that her WT was not long as the recruitment process served to occupy her whilst she waited for the midwife consultation:

‘But it would have been that shorter because they also started late. So, in the meantime when I was in the study the time was flowing also. So, when I came back everything was going a bit faster.’ (Participant 014, 19, initial visit)

‘The recruitment process for the study did extend their clinic visits. The one participant was seen last as a result of the recruitment process. The other participant was third in line to see the midwife. When she arrived back from the recruitment process, there were only three patients left in the clinic.’ (Researcher’s observation, female, midwife)

## Discussion

The WT within the antenatal clinic was long for both the initial and follow-up visits. The factors that contributed towards the LWT were related to staff factors, patient factors, operational functioning, communication, equipment and infrastructure and research studies in the facility.

### Waiting times within the antenatal care

The WTs were longer than the recommended 2-h waiting period for South Africa. The follow-up appointments’ WTs are similar to other developing countries such as Nigeria, where the average WT was 237.6 min (Okonofua et al. 2018:11). However, the initial visit’s WTs were much longer and were because of the follow-up appointments that were seen first, irrespective of their arrival times. This finding is congruent with the findings of another study that found that illogical queuing and not attending to patients in the order that they arrived significantly affect individual WTs (Osundina & Opeke 2017:9).

Although many studies have been conducted on WTs, the factors that influence WTs are often context specific and, therefore, there are no consistent WTs on which to benchmark.

### Barriers to waiting times

Patient flow increased the WTs and was attributed to poor queuing system, lack of signage and ineffective orientation of patients. The lack of patient flow is consistent with other research, which stated that 41.6% of healthcare facilities did not have a patient flow system which contributed to increased WT (Valla 2016:37). Improving patient flow can improve operational efficiency and has a positive effect on patient satisfaction, which could improve utilisation of the service (Osundina & Opeke 2017:7).

Lack of equipment such as baumanometers and telephones and poor infrastructure increased the WTs. The culmination of these factors resulted in bottle necks in the patient flow. To avoid bottle necks, women would go to other service points and this created a disorganised patient flow where service points were missed. This compounded the delay with the ANC activities and increased WTs. The negative influence of a lack of equipment on patient flow was echoed in another (Osundina & Opeke 2017:9). Increasing the amount of equipment and the human resources to use the equipment can have a positive influence on WTs.

The work activities were different for an initial visit than for a follow-up visit. Generally, follow-up appointments had fewer ANC activities, and this resulted in shorter WTs. Compounding this, the follow-up visits were seen before initial visits that lengthened the WTs of the initial visits. Similar findings were found in another study where new patients and unscheduled patients spent more time at the health facility (Colebunders et al. 2007:150). This indicated increased work activities; therefore, the patients spend more time at the facility as opposed to follow-up appointments.

Patients’ acuity levels increased WTs. The presence of infection, hypertensive disorders, HIV and minor ailments of pregnancy are unpredictable, and the management thereof lengthens the consultation and increases WT of the following person. The lack of telephones and infrastructure logistics further compounded the WTs. The relationship with patient acuity was echoed in another study that found that patient acuity, related to priority, resulted in a delay with the after coming patients (Zafar et al. 2016:1191). Antenatal care’s service is a response-based service and therefore patient acuity is an unavoidable factor where WTs are concerned.

The referral system with regard to availability of the physician, telephone lines that are engaged and physicians confirming management with senior consultants created a delay with the midwives’ consultation. As a result, the after coming patients experienced LWTs. This was confirmed by another study that found that poor information handover between colleagues resulted in patient delays (Naiker et al. 2018).

Duplicate documentation by the midwives increased the length of the consultation times and contributed to LWTs. The duplicate documentation was in response to women losing their MCRs, which influenced the continuity of care. Suggestions made by the staff were to photocopy the original document; however, this was rejected. A reason for this could be the cost implications. Record keeping has been previously cited as a cause of LWT (Mutshatshi et al. 2018:3). Medico-legally, accurate and comprehensive record keeping is imperative and should be viewed as an unavoidable barrier to LWT.

Student midwives in the antenatal clinic increased WTs. The midwives needed to teach the students, which was done through explanations, demonstrations and confirming the findings of the students. The teaching responsibilities of the midwives increased the consultation times. Another study confirmed that training students increased WTs (Motloba et al. 2018:401). Teaching is one of the responsibilities of the midwives; failure to teach and allow the students to practice can result in poor competence of the graduate. Therefore, this should be viewed as an unavoidable factor to LWTs.

The predominant language spoken in the clinic was Xhosa. As a result, women who did not understand Xhosa experienced delays with their ANC. This hindered patient flow and resulted in the ANC activities not being done. The influence of language barriers on WTs was not found in the literature. However, effective communication was found to increase satisfaction, despite participants experiencing LWTs. This reiterates the need for effective communication amongst healthcare personnel. Therefore, language barriers are an avoidable cause of LWTs.

### Limitations of the study

The sample for the midwives was small and this compromised the data that could have been obtained.

### Recommendations

The recommendations are to place signage in the clinic to indicate the patient flow, establishing a numbering system to avoid illogical queuing, attending to initial visits and follow-up visits on separate days, employing an interpreter for the foreign national women, using English as the primary language in the clinic and the acquisition of more telephones and baumanometers.

## Conclusion

The factors that influence WTs were interrelated and varied daily. The main reasons for LWTs included staff factors, patient factors, operational factors, communication, equipment and infrastructure and research participant recruitment. The barriers to WTs were unpredictable and difficult to control. The results of the study can be used to inform WT guidelines and improve WTs in the antenatal clinic.
